# Surgery for elderly patients with resectable pancreatic cancer, a comparison with non-surgical treatments: a retrospective study outcomes of resectable pancreatic cancer

**DOI:** 10.1186/s12885-019-6255-3

**Published:** 2019-11-12

**Authors:** Hyeong Min Park, Sang-Jae Park, Sung-Sik Han, Seoung Hoon Kim

**Affiliations:** Department of Surgery, Center for Liver and Pancreatobiliary Cancer, National Cancer Center, 323, Ilsan-ro, Ilsandong-gu, Goyang-si, Gyeonggi-do South Korea

**Keywords:** Pancreatic cancer, Elderly patient, Surgery, Non-surgical treatment

## Abstract

**Background:**

We designed a retrospective study to compare prognostic outcomes based on whether or not surgical resection was performed in elderly patients aged(≥75 years) with resectable pancreatic cancer.

**Methods:**

We retrospectively analyzed 49 patients with resectable pancreatic cancer (surgery group, resection was performed for 38 cases; no surgery group, resection was not performed for 11 cases) diagnosed from January 2003 to December 2014 at the National Cancer Center, Korea.

**Results:**

There was no significant difference in demographics between the two groups. The surgery group showed significantly better overall survival after diagnosis than the no surgery group (2-year survival rate, 40.7% vs. 0%; log-rank test, *p* = 0.015). Multivariate analysis revealed that not having undergone surgical resection [hazard ratio (HR) 2.412, *P* = 0.022] and a high Charlson comorbidity index (HR 5.252, *P* = 0.014) were independent prognostic factors for poor overall survival in elderly patients with early stage pancreatic cancer.

**Conclusions:**

In the present study, surgical resection resulted in better prognosis than non-surgical resection for elderly patients with resectable pancreatic cancer. Except for patients with a high Charlson comorbidity index, an aggressive surgical approach seems to be beneficial for elderly patients with resectable pancreatic cancer.

## Background

Pancreatic cancer is one of the most malignant diseases and its prognosis is dismal. In contrast to the steady increase in survival for most cancers, advances have been slow for pancreatic cancers, for which the 5-year relative survival is currently 8%. This low survival rate for which 5-year survival is 2% is partly due to the high rate of an advanced stage at the time of diagnosis [1].

Only complete resection of the lesion provides a chance at cure. However, the survival rates following surgery for pancreatic cancer remain poor and have improved only marginally in recent decades [2, 3]. In elderly patients, pancreatic cancer is associated with higher perioperative mortality and morbidity rates, higher requirement of an intensive care unit stay, increased length of hospital stay, and higher rates of hospital readmission after pancreatectomy [4–8]. Recently, in contrast to the existing theory, some authors assert justification of surgical resection for pancreatic cancer in selected elderly patients. Brahmbhat et al. reported that although elderly patients had a significantly higher postoperative morbidity rates compared with younger patients, age was not associated with an increased risk of 90-day mortality following pancreatic resection [9]. Furthermore, no difference in severe morbidity and mortality rates based on the age was reported a study by Okabayashi et al. [10].

Few studies have compared the relative benefits of elderly patients having and not having undergone surgical resection for pancreatic cancer.

To the best of our knowledge, there is no study comparing outcomes in elderly patients with resectable pancreatic cancer having and not having undergone surgical resection except the study by Marmor et al. reported that compared with chemotherapy, surgical resection is associated with a very small survival benefit in elderly patients (aged ≥80 years with lymph node metastasis) [11]. Thus, in this retrospective study, we compared prognostic results of undergoing vs. not undergoing surgical resection and identified factors related to prognosis in elderly patients with resectable pancreatic cancer.

## Methods

### Study population and data collection

In this retrospective study, we defined patients aged ≥75 years as the elderly. We reviewed the medical records of patients diagnosed with pancreatic cancer at the National Cancer Center in Korea between January 2003 and December 2014 to assess the impact of surgical resection on prognosis. Patients were stratified into two groups based on whether they underwent surgical resection or not. AJCC stage was clinically determined at the time of diagnosis based on pretreatment radiological imaging, including computed tomography (CT), magnetic resonance imaging (MRI) with or without positron emission tomography (PET). All patients enrolled in this study were operable if they (1) did not have severe comorbidities or cognitive impairment, (2) were expected to show extended prognosis following surgery, (3) had an acceptable functional status, and (4) had no advanced tumors, including distant metastasis. As a result, elderly patients with resectable pancreatic cancer (AJCC stage I or II) were enrolled in this study. The Eastern Cooperative Oncology Group (ECOG) performance status (PS) and the updated Charlson comorbidity indices system were available for all patients [12–14]. Because cancer is the disease of interest, it was not included in the Charlson comorbidity index. The chemotherapy regimen for resectable pancreatic cancer at our institution comprises gemcitabine-based therapy with or without the use of other drugs. The administration of chemotherapy was defined as completed at least 1 cycle of chemotherapy. The chemotherapy in the ‘Surgery group’ was all conducted as adjuvant therapy.

### Follow-up and assessment

Follow-up evaluation included tumor assessment by chest and abdominal-pelvic CT, tumor marker levels, and other laboratory tests (complete blood cell count, electrolytes, liver function test, and renal function test). Follow-up data were obtained via medical records or telephone contact up to December 2016. The primary end-point was overall survival (OS) in patients with resectable pancreatic cancer after diagnoses compared between the surgery and no surgery groups. The secondary end point of the study was identification of factors indicating survival. In addition, we evaluated the postoperative outcomes of patients who underwent surgical resection. Postoperative morbidities were defined according to the Clavien–Dindo (C-D) classification, and severe complications were limited to C-D classification III or IV [15]. Postoperative recurrence was determined based on CT and/or PET.

### Statistical analysis

The collected data were analyzed using IBM SPSS version 22.0 (IBM Corp., Armonk, NY, USA). OS was analyzed using the Kaplan-Meier method, and the survival difference between the surgery and no surgery groups was compared using the log-rank test. Differences between continuous variables were analyzed using student’s t-test. Categorical variables were compared using Pearson’s chi-square test. The cumulative survival rate and univariate analysis for survival were analyzed using the Kaplan–Meier method, and the log-rank test was used to compare significant differences. To investigate the combined effects of different variables on survival, Cox’s proportional hazards regression model was used. For all tests, a *p*-value of less than 0.05 was considered significant corresponding to a 95% confidence interval (95% CI).

## Results

### Patients

Among 1611 patients who were diagnosed with pancreatic cancer at the National Cancer Center in Korea between January 2003 and December 2014, the number of elderly patients (aged ≥75 years) was 290. Patients with advanced pancreatic cancer (*n* = 234) were excluded. Furthermore, patients with simultaneous diagnosis of another malignant disease (*n* = 3) or a history of another malignant disease (*n* = 4) were excluded. Finally, 49 patients with resectable pancreatic cancer were enrolled in this study. Thirthy-eight patients who underwent surgical resection for pancreatic cancer were categorized in the surgery group and 11 patients who did not undergo surgical resection were categorized in the no surgery group. There were 32 men and 17 women with a median age of 78 years (range: 75–87 years). In the surgery group, a conventional Whipple’s operation (PD) was performed in 6 (12.2%) patients, pylorus-preserving pancreaticoduodenectomy (PPPD) in 16 (32.7%) patients, total pancreatectomy (TP) in 4 (8.2%) patients, and distal pancreatectomy (DP) in 12 (24.5%) patients. Among the 11 patients in the no surgery group, 3 patients could not receive surgery due to a poor generalized state of health and 8 patients declined surgery or any other form of treatment. The median follow-up interval for all patients in the study (*n* = 49) was 12 months (range: 2–95 months), and the median survival time was 16 ± 2.9 months. The 1-year, 3-year, and 5-year survival rates were 58.5, 20.2, and 10.1%, respectively.

### Comparisons of clinicopathological factors between the surgery group and no surgery groups

**Fig. 1:**
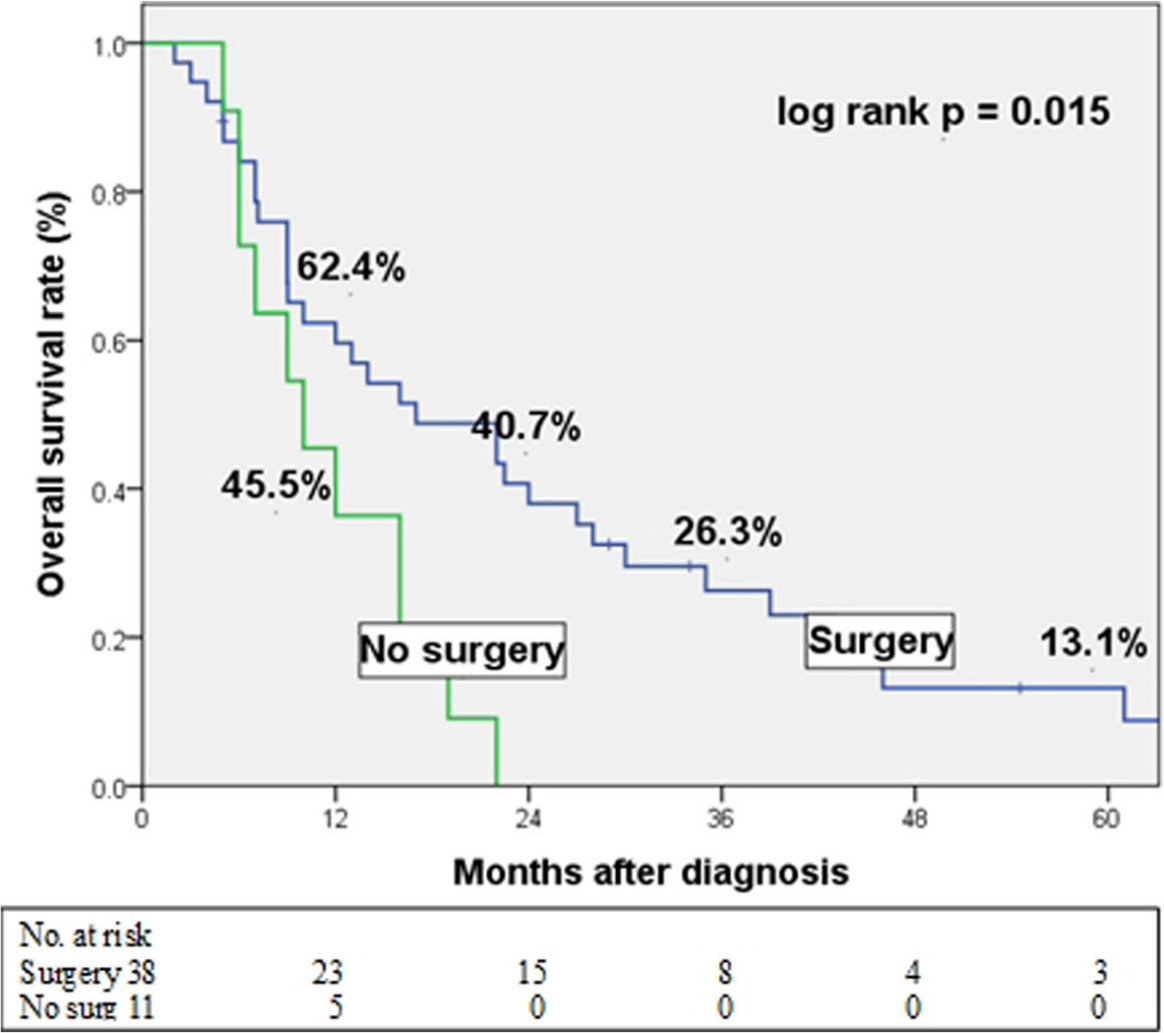
Kaplan-Meier estimates for overall survival in elderly patients with resectable pancreatic cancer. Patients who underwent surgical resection had a better survival than those who did not undergo surgical resection (*p* = 0.015)

Several clinicopathological factors were analyzed to compare differences between surgery group (*n* = 38) and no surgery group (*n* = 11; Table 1). There was no significant difference in any factors analyzed. When we compared the OS rate, the surgery group showed a better prognosis than the no surgery group (log-rank test, *p* = 0.015; Fig. 1). The median survival time after diagnosis was 17 ± 5.5 months and 10 ± 2.8 months for the surgery and no surgery groups, respectively. Overall, 24/38 (65.8%) patients in the surgery group died due to tumor progression (vs. 11/11 in the no surgery group). The remaining 10/38 patients died as a result of septic shock caused by bile peritonitis occurring after surgery (*n* = 1), severe pneumonia (*n* = 3) and an unknown cause without any clinical evidence of recurrence or progression of tumor (*n* = 6; Table 2).

**Table 1:** Characteristics of patients with early stage pancreatic cancer (*N* = 49)

Factors	Surgery (*N* = 38)	No surgery (*N* = 11)	*P* value
Sex			.55
Male	24 (63.2%)	8 (72.7%)	
Female	14 (36.8%)	3 (27.3%)	
Age (mean, range)	78 (75–87)	80 (75–86)	.07
BMI (mean, range)	21.8 (17.5–26.8)	22.0 (17.4–25.4)	.81
Smoking			.06
Yes	9 (23.7%)	5 (55.6%)	
No	29 (76.3%)	4 (44.4%)	
Alcohol			.46
Yes	12 (31.6%)	4 (44.4%)	
No	26 (68.4%)	5 (55.6%)	
HTN			1.00
Yes	19 (50%)	5 (50%)	
No	19 (50%)	5 (50%)	
DM			.25
Yes	19 (50%)	3 (30%)	
No	19 (50%)	7 (70%)	
Charlson comorbidity Index			.92
0	16 (42.1%)	5 (45.5%)	
1	14 (36.8%)	4 (36.4%)	
2	6 (15.8%)	1 (9.1%)	
3	2 (5.3%)	1 (9.1%)	
ECOG PS			.19
0	9 (23.7%)	2 (18.2%)	
1	26 (68.4%)	6 (54.5%)	
2	3 (7.9%)	2 (18.2%)	
3	0	1 (9.1%)	
Family Hx. of malignancy			.12
Yes	7 (18.4%)	0	
No	31 (81.6%)	11 (100%)	
Location of tumor			.85
Head	26 (68.4%)	8 (72.7%)	
Body & Tail	12 (31.6%)	3 (27.3%)	
Preoperative data (median ± SD)			
Hb (g/dL)	12.5 ± 1.5	13.0 ± 2.0	.46
INR	1.04 ± 0.19	1.10 ± 0.12	.57
T. Bil	0.8 ± 3.3	0.7 ± 1.1	.17
Albumin (g/dL)	4.1 ± 0.4	4.1 ± 0.4	.54
CA 19–9 (U/mL)	175.0 ± 713.8	169.2 ± 663.2	.98
TNM Staging			.32
1	3 (7.9%)	2 (18.2%)	
2	35 (92.1%)	9 (81.8%)	
CTx. and/or RTx.			.95
Yes	10 (26.3%)	3 (27.3%)	
No	28 (73.7%)	8 (72.7%)	

*BMI* body mass index, *ECOG* Eastern Cooperative Oncology Group, *PS* performance status, *Hx.* history, *SD* standard deviation, *Hb* hemoglobin, *INR* international normalized ratio, *T. Bil* total bilirubin, *CA 19–9* carbohydrate antigen 19–9, *CTx.* chemotherapy, *RTx.* radiotherapy

**Table 2:** Causes of death of patients with early stage pancreatic cancer

Causes of death	Surgery (*N* = 38)	No surgery (*N* = 11)	*P* value
			.18
Not dead	4 (10.5%)	0	
Operative mortality	1 (2.6%)	0	
Tumor progression	24 (65.8%)	11 (100%)	
Other causes	9 (26.4%)	0	

### Identification of factors for survival

In univariate analysis, surgical resection (log-rank test, *p* = 0.015) and Charlson comorbidity index (log-rank test, *p* = 0.044) showed significance on OS (Table 3). And these two factors, not having undergone surgical resection [hazard ratio (HR) 2.412, *P* = 0.022] and high Charlson comorbidity index (HR 5.252, *P* = 0.014) were independent prognostic factors for poor OS in elderly patients with resectable pancreatic cancer (Table 4).

**Table 3:** Univariate analysis of factors for overall survival in patients with early stage pancreatic cancer, Kaplan-Meier method

Factors	No. of patients (%)	Median OS [95% CI]	*P* value
Sex			.11
Male	32 (65.3%)	12.0 [4.4–19.6]	
Female	17 (34.7%)	22.0 [10.1–33.9]	
Age			.47
75≥, and < 80	38 (77.6%)	16.0 [10.0–22.0]	
≥ 80	11 (22.4%)	10.0 [5.5–14.5]	
BMI			.57
< 25	43 (87.8%)	13.0 [7.5–18.5]	
≥ 25	6 (12.2%)	19.0 [9.4–28.6]	
Smoking			.32
Yes	14 (29.8%)	13.0 [5.2–20.8]	
No	33 (70.2%)	16.0 [10.2–21.8]	
Alcohol			.33
Yes	16 (34%)	12.0 [0.3–23.8]	
No	31 (66%)	16.0 [10.7–21.3]	
HTN			.74
Yes	24 (50%)	12.0 [6.0–18.0]	
No	24 (50%)	17.0 [6.1–27.9]	
DM			.58
Yes	22 (45.8%)	9.0 [5.2–12.8]	
No	26 (54.2%)	10.9–23.1]	
Charlson comorbidity Index			.04
0	21 (42.9%)	19.0 [12.5–25.5]	
1	18 (36.7%)	9.0 [0.7–17.3]	
2	7 (14.3%)	16.0 [5.7–26.3]	
3	3 (6.1%)	6.0 [4.4–7.6]	
ECOG			.18
0	11 (22.4%)	22.0 [12.6–31.4]	
1	32 (65.3%)	14.0 [7.3–20.7]	
2	4 (8.2%)	5.0 [2.1–7.9]	
3	2 (4.1%)	6.0	
Family hx. of malignancy			.27
Yes	7 (14.3%)	9.0 [6.4–11.6]	
No	42 (85.7%)	16.0 [8.5–23.5]	
Location of tumor			.84
Head		12.0 [6.3–17.7]	
Body & Tail		19.0 [2.6–35.4]	
Hb (g/dL)			.59
≥ 13	19 (38.8%)	16.0 [6.2–25.8]	
< 13	30 (61.2%)	13.0 [2.5–23.5]	
PT (INR)			.10
≤ 1.2	43 (93.5%)	16.0 [8.6–23.4]	
> 1.2	3 (6.5%)	9.0 [4.2–13.8]	
T. Bil			.92
≤ 1.2	32 (65.3%)	16.0 [6.3–25.7]	
> 1.2	17 (34.7%)	14.0 [10.2–17.9]	
Albumin (g/dL)			.55
≥ 3.3	45 (91.8%)	16.0 [10.8–21.2]	
< 3.3	4 (8.2%)	12.0 [0.0–24.0]	
CA 19–9 (U/mL)			.38
≤ 37	14 (29.2%)	10.0 [8.3–11.8]	
> 37	34 (70.8%)	16.0 [10.4–21.6]	
Stage			.12
1	5 (10.2%)	22.5 [15.0–30.0]	
2	44 (89.8%)	13.0 [7.5–18.5]	
Surgical resection			.02
Yes	38 (77.6%)	16.0 [5.1–26.9]	
No	11 (22.4%)	10.0 [4.6–15.4]	
CTx. and/or RTx.			.09
Yes	17 (34.7%)	22.0 [8.6–35.4]	
No	32 (65.3%)	10.0 [4.6–15.4]	

*No*. number, *OS* overall survival, *CI* confidence interval, *BMI* body mass index, *ECOG* Eastern Cooperative Oncology Group, *PS* performance status, *Hx.* history, *SD* standard deviation, *Hb* hemoglobin, *INR* international normalized ratio, *T. Bil* total bilirubin, *CA 19–9* carbohydrate antigen 19–9, *CTx.* chemotherapy, *RTx.* radiotherapy

**Table 4:** Multivariate analysis for overall survival of patients with early stage pancreatic cancer

Factors	*P*-value	OR	95.0% CI	
			Min.	Max.
Surgical resection	.02			
Yes		1	Reference	Reference
No		2.41	1.13	5.11
Charlson comorbidity Index	.10			
0		1	Reference	Reference
1	.48	1.28	0.63	2.57
2	.94	1.03	0.42	2.49
3	.01	5.25	1.39	19.76

*OR* odds ratio, *Min.* minimum, *Max.* maximum

### Outcomes of patients who underwent surgery

Among elderly patients with resectable pancreatic cancer, recurrences occurred in 27 (71.1%) patients following surgery. The median disease-free survival times were 14 months (range, 1–103 months) in the surgery groups, respectively. The median survival times were 22 months and 14 months (range, 2–95 months), respectively. Severe postoperative complications (C-D classification III/IV) occurred in 12 (31.6%) patients. Among the 4 patients who were alive at the last follow-up, only one patient had remained without disease for 95 months. Two had remained alive for 52 months and 27 months with recurrence in the liver detected at postoperative months 36 and 4, respectively. Lastly, one patient had remained alive for 31 months with peritoneal seeding, identified postoperative month 20.

## Discussion

Although surgical resection is the only curative treatment modality for pancreatic cancer, many elderly patients are not good candidates for surgery or decline surgery. Furthermore, they are less likely to receive other treatments including chemotherapy, compared with younger patients [16–18]. Because the population is aging, it is estimated that the number of elderly patients with pancreatic cancer will continue to rise [19]. In addition, life expectancy is increasing. In the general population, the average life expectancy of an octogenarian is 7.44 years for males and 9.23 years for females according to the 2010 life-tables of the United States Social Security Administration [20]. It means that the number of elderly people can expect to live fairly longer. With advancements in surgical skills, devices, and perioperative management, the prognoses of many malignant diseases are improving. The prognosis of pancreatic cancer following various treatment modalities has also improved, although the degree of improvement is marginal [21]. Some authors reported worse prognosis among elderly patients following surgery compared with that among younger patients [22–24]. When we analyzed our data for overall patients who underwent surgery for resectable pancreatic cancer, we found that the median OS of elderly patients was significantly shorter than that of younger patients (16.0 vs. 23.4 months, *p* = 0.010, data not shown). However, this result should not be considered as evidence for contraindicating surgery in elderly patients with pancreatic cancer because the life expectancy between the two age groups may be different. In this study, only patients with age more than 75 years were included. Many of the patients in the study were under the age of 80 years, so we analyzed the association between age and overall survival based on age 80 years. However, age was not a factor associated with overall survival in patients with resectable pancreatic cancer.

Recently, some authors have reported that elderly patients who underwent surgical resection for pancreatic cancer showed outcomes comparable to those shown by younger patients [19, 25, 26].

As discussed above, the benefits of surgical resection for pancreatic cancer in elderly patients are controversial. Therefore, comparing the outcomes of elderly patients based on whether surgical resection was undertaken or not may be more suitable to identify the benefits of surgery in elderly patients than comparing age groups. However, there have been few studies comparing the prognosis in elderly patients with resectable pancreatic cancer between surgery and no surgery groups. In a study reported by He et al., patients who underwent surgery for resectable pancreatic cancer had a significantly higher 5-year OS rate (25.0 vs 2.3%; *P* < 0.0001) and a higher median survival time (24.3 vs 5.8 months) compared with patients who did not undergo surgery [27]. This result gives more weight to the assertion that age alone should not be a contraindication to surgery in elderly patients with pancreatic cancer. Clinicians need to more formally assess the individual operability in terms of comorbidities, cognitive status, preoperative functional status, and frailty [28]. For patients and their relatives, it is important that they understand the risk of mortality, complications, and need for skilled nursing care after pancreatic surgery when making this decision, but it is equally important that they understand the benefit of surgical resection and that this benefit is not significantly diminished with increasing age despite the greater short-term complications [29].

We identified that surgical resection and Charlson comorbidity index were independent risk factors for OS in elderly patients with resectable pancreatic cancer. Surgical resection is well-known as the only curative treatment modality for resectable pancreatic cancer. The relation between a high Charlson comorbidity index and poor prognosis is supported by a study reported by Asano et al. which showed that the OS rate was significantly higher in the low Charlson age comorbidity index group than in the high Charson comorbidity index group (*P* = 0.047), and a high Charlson age comorbidity index was an independent factor of poor survival (*P* = 0.024) [30].

This study has some limitations. First, as with all retrospective studies, there may have been a selection bias regarding the diagnosis and treatment of patients with pancreatic cancer. Quality of life, another important outcome of patients with pancreatic cancer could not be evaluated due to the retrospective nature of this study. In addition, the number of cases was small despite a study period of > 10 years, reflecting the fact that elderly patients with resectable pancreatic cancer are rare. For example, the distribution of the ECOG PS score was different between two groups in this study. However, there was no statistical significant difference. Although there was a just one patient with ECOG PS 3 in ‘No surgery group, it might affect poor outcome in ‘No surgery group’. There is a limitation to assessing the degree of the impact of this difference in small-scale study. Thus, meaningful statistical analysis was challenging, which may be overcome by performing a multicenter study in the future. We were also unable to compare the efficacy between multimodal and unimodal treatment of resectable pancreatic cancer with or without surgery because of the lack of evidence as to which treatment is better; this is possibly because it depends on the clinical state of each patient. Further studies are necessary to validate the various treatment modalities. Finally, despite the small number of patients, the strategy of treatment modalities applied was heterogeneous. This might be due to the poor prognosis with short survival time of pancreatic cancer and the various degrees of positivity pertaining to the treatment of patients.

## Conclusions

In conclusion, whether surgical resection was performed or not and the Charlson comorbidity index were independent risk factors for OS in elderly patients with resectable pancreatic cancer. Therefore, except for those with a high Charlson comorbidity index, aggressive surgical approach seems beneficial for elderly patients with resectable pancreatic cancer.

## Data Availability

The datasets used and/or analysed during the current study are available from the corresponding author on reasonable request.

## Abbreviations

CT: Computed Tomography
DP: Distal Pancreatectomy
ECOG: Eastern Cooperative Oncology Group
HR: Hazard Ratio
MRI: Magnetic Resonance Imaging
OS: Overall Survival
PD: Pancreaticoduodenectomy
PPPD: Pylorus Preserving Pancreaticoduodenectomy
PS: Performance Status
TP: Total Pancreatectomy
